# Endangered island endemic plants have vulnerable genomes

**DOI:** 10.1038/s42003-019-0490-7

**Published:** 2019-06-27

**Authors:** Tomoko Hamabata, Gohta Kinoshita, Kazuki Kurita, Ping-Lin Cao, Motomi Ito, Jin Murata, Yoshiteru Komaki, Yuji Isagi, Takashi Makino

**Affiliations:** 1 Graduate School of Life Sciences, Tohoku University, Aoba-ku, Sendai 980-8578 Japan; 2 Graduate School of Agriculture, Kyoto University, Sakyo-ku, Kyoto 606-8502 Japan; 3 Graduate School of Arts and Sciences, University of Tokyo, Meguro-ku, Tokyo 153-8902 Japan; 4 Koishikawa Botanical Garden, Graduate School of Science, University of Tokyo, Bunkyo-ku, Tokyo 112-0001 Japan

**Keywords:** Evolutionary ecology, Evolutionary genetics, Conservation biology, Plant genetics

## Abstract

Loss of genetic diversity is known to decrease the fitness of species and is a critical factor that increases extinction risk. However, there is little evidence for higher vulnerability and extinction risk in endangered species based on genomic differences between endangered and non-endangered species. This is true even in the case of functional loci, which are more likely to relate to the fitness of species than neutral loci. Here, we compared the genome-wide genetic diversity, proportion of duplicated genes (*P*_D_), and accumulation of deleterious variations of endangered island endemic (EIE) plants from four genera with those of their non-endangered (NE) widespread congeners. We focused on exhaustive sequences of expressed genes obtained by RNA sequencing. Most EIE species exhibited significantly lower genetic diversity and *P*_D_ than NE species. Additionally, all endangered species accumulated deleterious variations. Our findings provide new insights into the genomic traits of EIE species.

## Abstract (web summary)

Tomoko Hamabata et al. used transcriptomics to reveal lack of genome-wide genetic diversity and a significant accumulation of deleterious genes in the endangered species of oceanic island. These genomic changes may affect overall plant fitness and potentially contribute to extinction risks of these isolated species.

## Introduction

As the drastic loss of species progresses worldwide^1^, ecological and biological studies that aim to minimize such losses and maximize conservation efficiency are urgently required. In general, endangered species are often characterized by small and isolated populations, and are therefore prone to loss of genetic diversity and inbreeding owing to larger effects of genetic drift^2,3^. In addition, the bottlenecks associated with the population decline of an endangered species facilitate the accumulation of deleterious alleles owing to the reduced efficacy of selection^4–6^. The cumulative effects of homozygosity for deleterious mutations at many loci can cause inbreeding depression. Further, the loss of genetic diversity can reduce the ability of the organisms to evolve in response to environmental change^7^. The genetic degradation resulting from small population sizes and isolation are particularly serious on islands, which are centers of small isolated populations and have high levels of endemism^8^. Although islands contribute to global biodiversity, insular species—particularly, oceanic island populations derived from a single individual in self-fertilizing species or a pair in non-self-fertilizing species—are generally expected to display low genetic diversity because of founder effects and lower subsequent population size^9^. Such species have few chances to recover genetic diversity owing to low immigration and gene flow in small, isolated populations on islands. In fact, insular species are known to have a higher risk of extinction^10^ and many existing island endemic species are classified as endangered^11^. However, there is little evidence that endangered island endemic (EIE) species experience the expected genomic changes: loss of genome-wide genetic diversity, or accumulation of deleterious mutations.

In conservation genetics, nucleotide diversity, number of alleles, and heterozygosity of neutral genetic markers (e.g., simple sequence repeats) have been broadly examined as aspects of genetic diversity. This is based on the general belief that the diversity of neutral loci should reflect the effective population size, and is expected to correlate with population fitness. Microsatellite-based multilocus heterozygosity is generally low in small populations^12^, and it is believed that this could represent the genome-wide heterozygosity at population level^13^. It is, however, unclear whether functional genetic variation—which seems to be directly linked to the fitness and viability of populations—can be affected by the same processes that have been demonstrated to affect such neutral genetic variations^14–16^. In addition, it is unclear if neutral markers are as useful for studying processes like local adaptation, loss of fitness by inbreeding, or the potential to adapt to changing environments, and because of this they are considered unsuitable for testing whether the issues of endangered species are a consequence of their genetics. To clarify which genetic factors contribute to vulnerability in EIE species, genome-wide genetic changes in functionality should be assessed.

High-throughput sequencing enables us to rapidly generate genome-wide data, and we can now handle these data using bioinformatic tools. Accessibility to genome-wide genetic variations, including the functional loci, will make it possible to directly examine whether small, isolated populations have typical genomic features, suggesting lowered adaptive potential compared with those of non-endangered (NE) species. RNA sequencing (RNA-seq), a high-throughput sequencing method often used for examining transcriptomes, provides the exhaustive sequences of expressed genetic coding regions in which non-synonymous sites are more likely to be under selection^17^. In addition, RNA-seq is a strong genome-wide approach to detect heterozygous variants^18^, although it may be difficult to call genotypes in loci with a low coverage of transcripts. RNA-seq can be applied to any species, regardless of whether there are reference genomic sequences of the target species, using de novo assembly. These characteristics of RNA-seq are beneficial in conservation genomics as they allow us to estimate exhaustive genetic diversity while selectively focusing on the functional genes, and may be useful to examine the genomic change that occurred in EIE.

The Ogasawara (Bonin) Islands constitute an archipelago located 1000 km south of the main islands of Japan and are registered as a UNESCO World Heritage site. Although the Ogasawara Islands contain characteristic biota, 37% of the plant taxa, including subspecies, are recognized as endemic. Various artificial changes to Ogasawara have degraded the native ecosystem, including the decline of pollinator communities^19,20^, collapse of insect populations and soil ecosystems by invasive animals^21,22^, severe predation of grasses and herbs by abandoned goats^23^, and disturbance of native vegetation by invasive plants^24,25^. At present, 66% of endemic plant species in the Ogasawara Islands are classified as endangered by the List of Threatened and Endangered Wildlife Species in Japan^26^. Although both in situ and ex situ conservation efforts for several endangered endemic species in Ogasawara have been conducted, in situ conservation is often considerably difficult owing to the high failure rate of seedlings^27^, and the regeneration of transplanted saplings has not yet been successful^23^. These situations suggest lowered fitness and serious vulnerability of these species in wild environments. However, the congeners of these endemic island species maintain stable populations on the main islands or continental area. These plant species provide an ideal opportunity to explore whether the observed vulnerability of EIE species is characteristically associated with low genome-wide genetic diversity in loci under natural selection. In addition, genomic features can be used to understand why the fitness of an EIE species is low compared to that of a NE species^28^.

In the present study, we examined an exhaustive list of expressed gene sequences using RNA-seq analysis, and explored whether EIE species generally exhibit a lower genetic diversity, even in genome-wide functional genes, than do NE congeners with wider distributions. As a basic index, we examined the numbers of synonymous heterozygous single-nucleotide variants (SNVs) that were putatively neutral^29^ and expected to reflect their effective population size. The efficacy of selection is expected to be reduced in small populations, and low selective pressure in small populations results in the accumulation of deleterious variation in functional loci^30^. We investigated whether the reduced efficacy of selection is evident in EIE species based on the proportion of non-synonymous SNVs in the total SNVs on the heterozygous loci of expressed genes. We additionally determined whether the supposed low selective pressure in EIE species populations actually resulted in the higher accumulation of deleterious amino acid variants in functional genes. Note that most non-synonymous mutations are deleterious or neutral^31^. In particular, the fixation probability of beneficial mutations is determined by population size, and therefore beneficial non-synonymous SNVs in endangered species with small population sizes are negligible^31^. To estimate the accumulation of deleterious amino-acid variants, we examined two different sites: the heterozygous non-synonymous SNVs within species, and the homozygous non-synonymous nucleotide substitutions between EIE and NE species. In addition, we examined the proportion of duplicated genes (*P*_D_) of each species, which was recently revealed to have a positive correlation with invasiveness in animals^32^ and adaptation to various environments in *Drosophila*^33^ and mammals^34^. It has been proposed that the maintenance of duplicated genes in a genome is enhanced by extensive environmental stimuli within the habitat ranges of species^33,34^, and thus EIE species, which generally inhabit restricted environments, are expected to possess less duplicated genes than widespread species. Accordingly, we examined the availability of *P*_D_ as a criterion of species vulnerability by comparisons between EIE and NE species using RNA-seq data and evidence genomic evolution to be lower *P*_D_ in range-restricted plant species. Finally, we discuss the availability of these estimates as indices for species vulnerability in conservation genomics based on our results.

## Results

### RNA sequencing

**Fig. 1:**
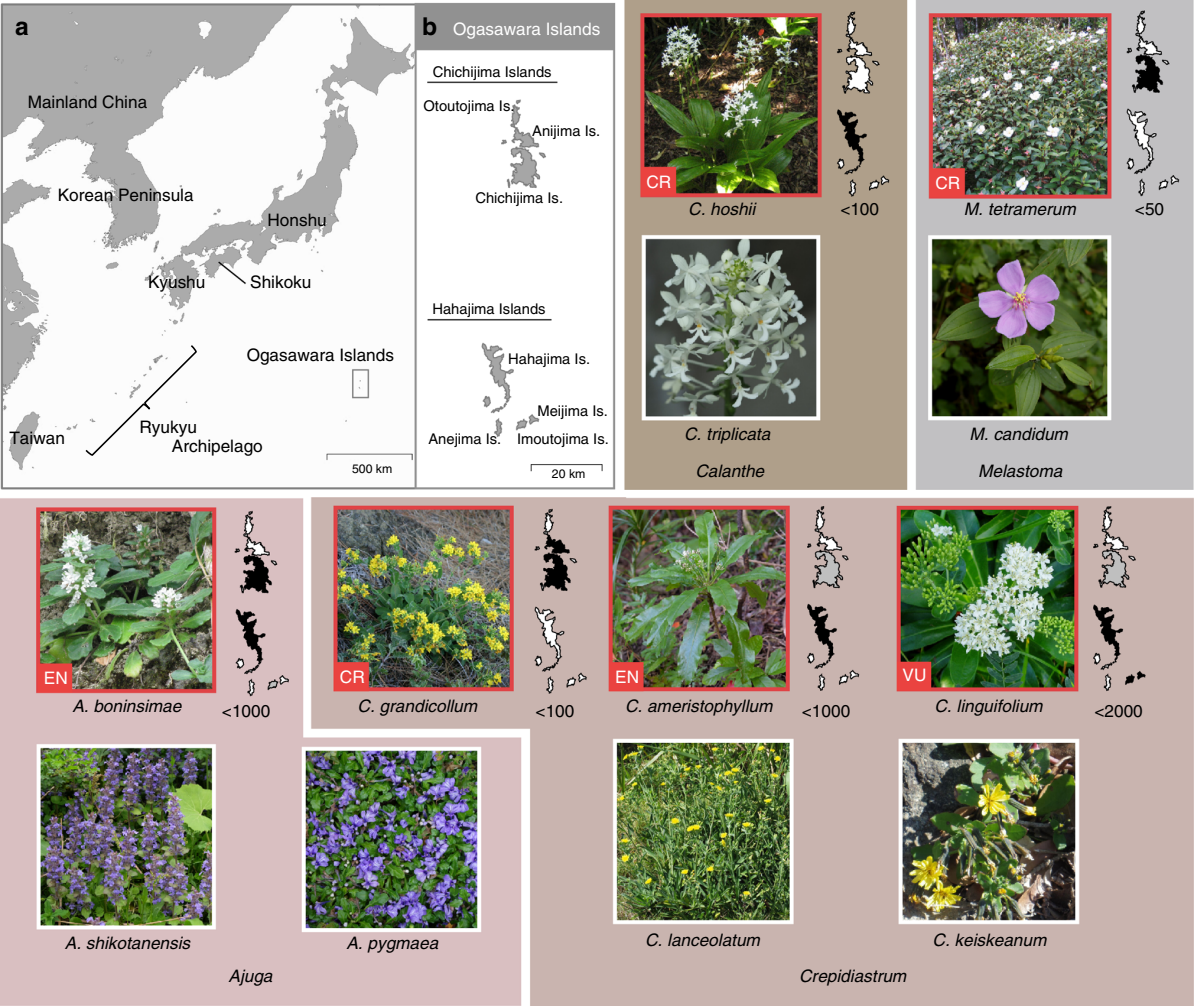
Map showing the distribution sites of the plant species used in this study **a**, and enlarged map of the Ogasawara Islands, where the six endangered plant species in this study are distributing **b**. Pictures boxed in red show the endangered island endemic (EIE) species with its classification in the Red List category (bottom left), and pictures boxed in white show the non-endangered (NE) species in each genus. Each painted island map on the right side of the picture of endangered species indicates its distribution; black-painted means distributing in the island, gray-painted means extinct in the island, and white-painted means not-distributing in the island. The number below the painted island map indicates the estimated current number of individuals of the endangered species. The Red List category is as follows; CR is Critically Endangered, EN is Endangered, and VU is Vulnerable. Distribution ranges of the non-endangered species are as follows; *A. pygmaea* in Kyushu and the Ryukyu Archipelago, *A shikotanensis* in Honshu, *C. lanceolatam* in Honshu, Korean Peninsula and Mainland China, *C. keiskeanum* in Honshu, Shikoku, Kyushu, Korean Peninsula and Mainland China, *C. triplicata* in Kyushu, the Ryukyu Archipelago, Taiwan, and Mainland China, and *M. candidum* the Ryukyu Archipelago, Taiwan, and Mainland China

To collect comparative data on genome-wide functional loci, we used RNA-seq with a de novo assembly. Samples for RNA extraction were collected from 15 individuals of six endemic plant species of four genera in Ogasawara: *Ajuga boninsimae* (*n* = 5), *Crepidiastrum grandicollum* (*n* = 4)*, Crepidiastrum ameristophyllum* (*n* = 1), *Crepidiastrum linguifolium* (*n* = 2)*, Calanthe hoshii* (*n* = 3), and *Melastoma tetramerum* (*n* = 3). These species are all classified as endangered by the List of Threatened and Endangered Wildlife Species of the Ministry of Environment, Japan, and were analyzed as EIE species in this study. The wild population sizes of each species are very small, and the level of gene flow among populations seems to be extremely low or non-existent owing to the extinction or decline of native pollinators^27,35^. For comparison, 17 individuals of one or two congener species with wider distribution ranges were selected from the correspondent genera: *Ajuga pygmaea* (*n* = 4), *Ajuga shikotanensis* (*n* = 3)*, Crepidiastrum lanceolatum* (*n* = 3), *Crepidiastrum keiskeanum* (*n* = 1), *Calanthe triplicata* (*n* = 3), and *Melastoma candidum* (*n* = 3), and were analyzed as NE species in this study. Information on each species is described in Fig. 1. RNA samples derived from single individuals of a species were sequenced separately on Illumina HiSeq 1000, HiSeq 2500, HiSeq 4000, or HiSeq X sequencers at 90, 100, or 150 nucleotide paired-end (PE) reads (Supplementary Data 1).

### Number of transcripts

The number of contigs reconstructed from the RNA-seq reads ranged from 72,590–168,631 in *Ajuga*, 80,825–151,228 in *Crepidiastrum*, 73,098–133,128 in *Calanthe*, and 84,713–127,261 in *Melastoma* (Supplementary Data 1). All short reads from all individuals of each species were used for *de novo* assembly also when multiple specimens were available. The assembly by mixed reads tended to reconstruct more contigs than individual assembly. However, there were no specific trends in the numbers of contigs, in the values of N50 and average lengths of contigs among sequencing lengths (90, 100, or 150PE) or among sequencing platforms (HiSeq version) (Supplementary Data 1).

### Genetic diversity by the numbers of heterozygous synonymous SNVs

**Fig. 2:**
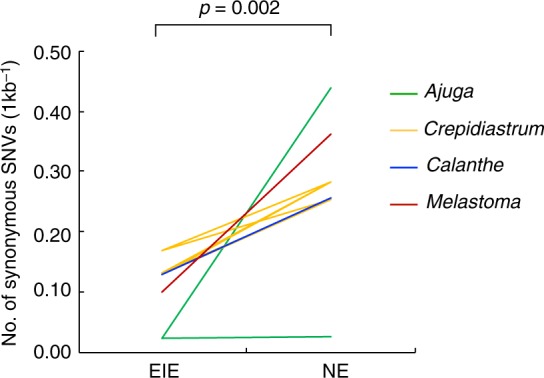
Mean values of the number of synonymous SNVs for heterozygous loci (counts per kb). The values compared between endangered island endemic (EIE) and non-endangered (NE) species within the same genus are connected by lines between species

Heterozygous SNVs in the coding regions of the longest transcript of each gene were counted and sorted as synonymous or non-synonymous. We estimated the genetic diversity of each sample based on the number of heterozygous synonymous SNVs in the longest coding sequences. Genetic diversity was evaluated by the counts per kb, and the mean values of all transcripts were compared. All EIE species exhibited lower genetic diversity than NE species (*p* = 0.002, exact binomial test, Fig. 2), although significance was not supported in some pairwise comparisons, i.e., between *A. boninsimae* and *A. pygmaea*, between *C. grandicollum* or *C. linguifolium*, and *C. lanceolatum*.

### The proportion of non-synonymous SNVs to total SNVs

The proportion of non-synonymous SNVs to total SNVs was also calculated for each longest transcript and the mean values between the EIE and NE species were compared. Overall, EIE species exhibited a higher proportion of non-synonymous SNVs to total SNVs than did NE species (*p* = 0.002, exact binomial test; Table 1, Supplementary Fig. 1). The high proportion of non-synonymous SNVs in EIE species were significantly supported in most pairwise comparisons except for a comparison between *A. pygmaea* and *A. boninsimae* (Table 2).

**Table 1:** Species category of Endangered island endemic (EIE) or Non-endangered (NE), proportions of duplicated genes (*P*_D_), and mean values of the number of synonymous SNVs at transcripts with heterozygous loci (the counts per kb), the proportion of non-synonymous SNVs to total SNVs on transcripts with heterozygous loci, the proportion of nonsense SNVs to total non-synonymous SNVs on transcripts with heterozygous loci, the proportion of deleterious variations in non-synonymous SNVs on transcripts with heterozygous loci estimated by PROVEAN and by SIFT

	EIE or NE	*P*_D_	Number of syn SNVs	Nonsyn SNVs/total SNVs	Nonsense SNVs/nonsyn SNVs	Proportion of deleterious variations in nonsyn SNVs	
						By PROVEAN	By SIFT
*Ajuga*							
*A. boninsimae*							
100PE	EIE	0.705	0.027	0.723	0.048	0.495	0.434
150PE-1	EIE	0.721	0.019	0.670	0.036	0.470	0.383
150PE-2	EIE	0.696	0.022	0.669	0.052	0.444	0.359
150PE-3	EIE	0.716	0.023	0.662	0.046	0.465	0.349
150PE-4	EIE	0.713	0.021	0.662	0.033	0.422	0.325
Average		0.710	0.022	0.677	0.043	0.459	0.370
*A. pygmaea*							
100PE	NE	0.715	0.025	0.699	0.040	0.464	0.389
150PE-1	NE	0.715	0.030	0.597	0.035	0.437	0.352
150PE-2	NE	0.730	0.024	0.686	0.043	0.397	0.344
150PE-3	NE	0.721	0.025	0.633	0.044	0.389	0.332
Average		0.720	0.026	0.654	0.041	0.422	0.354
*A. shikotanensis*							
100PE	NE	0.740	0.451	0.502	0.012	0.318	0.194
150PE-1	NE	0.734	0.414	0.512	0.023	0.320	0.208
150PE-2	NE	0.748	0.456	0.497	0.018	0.317	0.197
Average		0.741	0.440	0.503	0.017	0.319	0.200
*Crepidiastrum*							
*C. grandicollum*							
100PE	EIE	0.696	0.170	0.593	0.020	0.388	0.256
90PE	EIE	0.707	0.200	0.589	0.024	0.385	0.226
150PE-1	EIE	0.642	0.149	0.611	0.035	0.414	0.281
150PE-2	EIE	0.659	0.153	0.610	0.035	0.427	0.282
Average		0.676	0.168	0.601	0.029	0.404	0.261
*C. ameristophyllum*							
100PE	EIE	0.721	0.133	0.599	0.024	0.386	0.260
*C. linguifolium*							
90PE-1	EIE	0.679	0.128	0.577	0.026	0.377	0.231
90PE-2	EIE	0.696	0.130	0.608	0.033	0.377	0.257
Average		0.687	0.129	0.593	0.030	0.377	0.244
*C. lanceolatum*							
100PE	NE	0.730	0.344	0.486	0.015	0.275	0.184
150PE-1	NE	0.775	0.158	0.496	0.025	0.290	0.197
150PE-2	NE	0.771	0.260	0.488	0.021	0.269	0.188
Average		0.759	0.254	0.490	0.020	0.278	0.190
*C. keiskeanum*							
90PE	NE	0.717	0.283	0.512	0.017	0.320	0.202
*Calanthe*							
*C. hoshii*							
150PE-1	EIE	0.709	0.119	0.591	0.023	0.361	0.264
150PE-2	EIE	0.716	0.116	0.584	0.031	0.360	0.253
150PE-3	EIE	0.702	0.150	0.567	0.025	0.349	0.248
Average		0.709	0.128	0.581	0.026	0.357	0.255
*C. triplicata*							
150PE-1	NE	0.736	0.261	0.505	0.015	0.261	0.214
150PE-2	NE	0.758	0.262	0.508	0.025	0.260	0.206
150PE-3	NE	0.760	0.244	0.505	0.028	0.267	0.214
Average		0.752	0.256	0.506	0.023	0.263	0.211
*Melastoma*							
*M. tetramerum*							
150PE-1	EIE	0.695	0.101	0.585	0.018	0.381	0.261
150PE-2	EIE	0.702	0.100	0.588	0.017	0.369	0.248
150PE-3	EIE	0.704	0.099	0.614	0.022	0.378	0.255
Average		0.701	0.100	0.596	0.019	0.376	0.255
*M. candidum*							
150PE-1	NE	0.692	0.326	0.511	0.012	0.309	0.212
150PE-2	NE	0.721	0.329	0.474	0.013	0.286	0.207
150PE-3	NE	0.697	0.427	0.503	0.013	0.280	0.204
Average		0.704	0.361	0.496	0.013	0.292	0.208

**Table 2:** *P* values of *t* test compared the mean values of the *P*_D_, the number of synonymous SNVs at transcripts with heterozygous loci (the counts per kb), the proportion of non-synonymous SNVs to total SNVs on transcripts with heterozygous loci, the proportion of nonsense SNVs to total non-synonymous SNVs on transcripts with heterozygous loci, and proportion of deleterious variations in non-synonymous SNVs on transcripts with heterozygous loci estimated by PROVEAN and SIFT between endangered island endemic [EIE] and non-endangered [NE] species

	*A. boninsimae* [EIE]
*A. pygmaea* [NE]	
*P*_D_	0.131
Synonymous SNV	0.103
Nonsyn/Syn SNV	0.372
Loss of function	0.595
PROVEAN	0.115
SIFT	0.503
*A. shikotanensis* [NE]	
*P*_D_	**0.004**
Synonymous SNV	**<** **0.001**
Nonsyn/Syn SNV	**<** **0.001**
Loss of function	**0.003**
PROVEAN	**<** **0.001**
SIFT	**0.001**

	*C. grandicollum* [EIE]	*C. linguifolium* [EIE]
*C. lanceolatum* [NE]		
*P*_D_	**0.013**	**0.036**
Synonymous SNV	0.127	0.170
Nonsyn/Syn SNV	**<** **0.001**	**0.004**
Loss of function	0.064	0.093
PROVEAN	**<** **0.001**	**0.004**
SIFT	**0.006**	**0.015**

	*C. hoshii* [EIE]
*C. triplicata* [NE]	
*P*_D_	**0.008**
Synonymous SNV	**<** **0.001**
Nonsyn/Syn SNV	**<** **0.001**
Loss of function	0.471
PROVEAN	**<** **0.001**
SIFT	**0.003**

	*M. tetramerum* [EIE]
*M. candidum* [NE]	
*P*_D_	0.765
Synonymous SNV	**0.002**
Nonsyn/Syn SNV	**0.016**
Loss of function	**0.048**
PROVEAN	**0.005**
SIFT	**0.001**

### Accumulation of deleterious amino acid variants in heterozygous sites

**Fig. 3:**
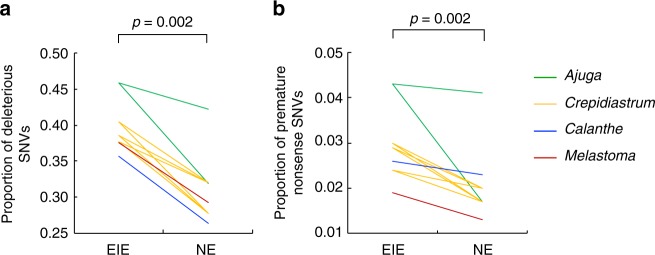
Mean values of the proportion of deleterious variations in non-synonymous SNVs on transcripts with heterozygous loci estimated by PROVEAN **a**, and the proportions of SNVs that varied by nonsense SNVs (loss-of-function SNVs) to total non-synonymous SNVs **b**. The values compared between EIE and NE species within the same genus are connected by lines between species

The non-synonymous SNVs were examined to determine whether each amino acid variation might affect protein function using Protein Variant Effect Analyzer (PROVEAN)^36^ and Sorting Intolerant From Tolerant (SIFT)^37^. In this analysis, we used only the transcripts sharing homology with proteins of the 39 angiospermous species whose genomes were registered in EnsemblPlants after identified by Protein Basic Local Alignment Search Tool (BLASTP) search. Both PROVEAN and SIFT predicted that all EIE species accumulated more variants that were supposedly deleterious to protein functions than did NE species (Fig. 3a, Supplementary Fig. 2, Table 1). Overall significance in accumulation of deleterious non-synonymous variations was supported in both methods (*p* = 0.002, exact binomial test). In pairwise comparisons, the NE species of *A. pygmaea* exceptionally represented comparable levels of accumulation of deleterious variants to the endangered *A. boninsimae* (*p* = 0.115 in PROVEAN and *p* = 0.503 in SIFT; Table 2). Furthermore, the proportion of nonsense SNVs to the total non-synonymous SNVs was significantly higher in EIE species of all four genera, suggesting more genes lost the function in EIE species (*p* = 0.002, exact binomial test; Table 1, Fig. 3b).

### Accumulation of deleterious amino-acid substitutions after speciation

To investigate the degree of accumulated deleterious substitutions during evolution, we focused on species-specific homozygous alleles, in which closely related species possessed different homozygous alleles. In this analysis, we reconstructed a fresh integrated set of transcripts for each species using all short reads derived from multiple specimens. Orthologous gene transcripts between each EIE and NE species were identified based on the longest coding sequences of the same gene ID by the BLASTP search. Only the transcripts sharing homology with proteins of the 39 angiospermous species in EnsemblPlants were examined. The proportions of deleterious substitutions on orthologous genes were, in all cases, significantly higher in EIE species than in NE species (*p* < 0.001 in PROVEAN and *p* < 0.001 in SIFT, exact binomial test; Supplementary Table 1, Supplementary Fig. 3).

### Proportion of duplicated genes

We carried out BLASTP search among transcripts and against proteins of the 39 angiospermous species whose genomes were registered in EnsemblPlants. Transcripts sharing homology with other transcripts and the 39 angiospermous species were defined as duplicated genes. In contrast, transcripts without any homology with other transcripts, but sharing homology with the 39 angiospermous species were identified as singleton genes. *P*_D_ was defined as the proportion of the number of duplicated genes to the total number of genes.

**Fig. 4:**
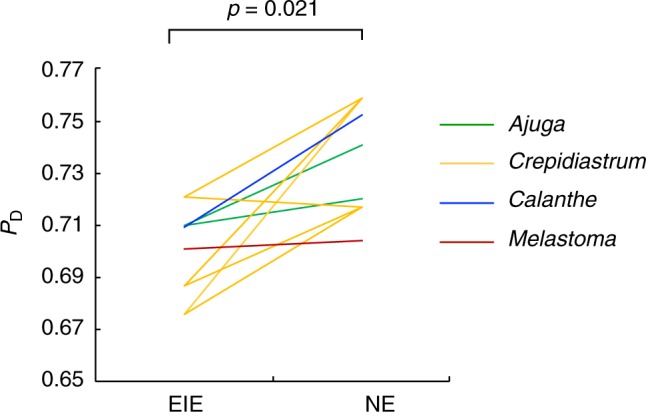
Proportion of duplicated genes for endangered and non-endangered species. The values compared between EIE and NE species within the same genus are connected by lines between species

The estimates of *P*_D_ presented lower values for EIE species than NE species in most cases (*p* = 0.021, exact binomial test; Fig. 4). However, the opposite trend was observed between *C. ameristophyllum* (*P*_D_ = 0.721) and *C. keiskeanum* (*P*_D_ = 0.717), and the values in endangered *A. boninsimae* and *M. tetramerum* were not significantly lower than *A. pygmaea* and *M. candidum*, respectively.

There is a possibility that the number of heterozygous SNVs may be artifactually inflated on paralogous transcripts owing to the difficulty in mapping. As we showed that *P*_D_ of NE species was likely to be higher than that of EIE species, the difference in *P*_D_ between EIE and NE species might influence our results. Therefore, we examined whether the trends in the numbers of heterozygous synonymous SNVs, the proportion of non-synonymous SNVs to total SNVs, accumulation of deleterious amino acid variants in heterozygous sites, and the proportion of nonsense SNVs to the total non-synonymous SNVs between EIE and NE were supported, when the transcripts derived from the duplicate genes were excluded. As a result, lower genetic diversity, higher proportions of non-synonymous SNVs, and more deleterious non-synonymous variations in EIE species did not change (Supplementary Data 2). In some comparisons, the difference between EIE and NE species were emphasized; endangered *A. boninsimae* accumulated significantly more deleterious non-synonymous variations on non-duplicated genes than non-endangered *A. pygmaea* (*p* = 0.018), although it was not significant when the transcripts from duplicated genes were included (Supplementary Table 2).

## Discussion

Genetic diversity between EIE and NE species was compared using the mean number of heterozygous synonymous SNVs based on their genome-wide coding regions per kb. This value is expected to reflect the effective population size (*N*e). EIE species generally exhibited lower genetic diversity, suggesting that the populations affected by bottleneck and founder effects. However, one specimen of non-endangered *C. lanceolatum* showed extremely low genetic diversity compared with the other two specimens. In addition, there was no significant difference in genetic diversity between endangered *A. boninsimae* and non-endangered *A. pygmaea*. Interestingly, these two species of genus *Ajuga* and one specimen of *C. lanceolatum* all showed a low percentage of transcripts with heterozygous loci: 3.0–5.1% in *A. boninsimae*; 3.7–4.5% in *A. pygmaea*, and; 10.7% in *C. lanceolatum* compared to that of *A. shikotanensis* (17.0–18.4%) or the other specimens of *C. lanceolatum* (15.6–20.4%; Supplementary Data 1). The observed results from these pairs and specimen suggest that their gene flow was limited. Indeed, it is known that *A. boninsimae* is self-compatible and *C. lanceolatum* is often cultivated in home gardens. Isolated populations of *A. boninsimae* might reproduce with a high frequency of self-fertilization. Self-fertilization increases homozygosity and decreases *N*_e_^38^. Although the breeding system of *A. pygmaea* is unknown, relatively low percentages of transcripts with heterozygous loci suggest that it might also reproduce by selfing. Also, the isolation of home gardens may cause limited gene flow, increase homozygosity, and reduce synonymous SNVs. Although the actual situations that these species and specimens experienced at reproduction are unknown, the present results strongly suggest that the genetic diversity estimated from the neutral loci could easily decrease even in NE widespread species.

The expectation that oceanic island species and populations have lower levels of genetic variation than their main island counterparts has been empirically evidenced in various taxa^9,30,39,40^. However, several recent studies have discovered examples in which oceanic plants could have a higher genetic diversity than their continental relatives, owing to the role islands play as climatic refugia during glacial periods^41,42^ and repeated colonization of oceanic plants to islands^43^. There is a growing belief that low genetic diversity in island populations cannot always be generalized^44^, and genetic diversity is more likely to be strongly influenced by population size and historical factors, such as the time since the population was established and past bottlenecks^42^. The present results suggesting that the genome-wide genetic diversity in functional loci was usually lower in island species than in widespread relatives was likely influenced by founder effects and low gene flow in small populations of oceanic islands. However, ecological traits such as the breeding system seem to more critically affect the level of genetic diversity than insularity. Although examination of variation in neutral loci is a classical and more common method for estimating the genetic diversity of species and populations, our results suggest that the neutral loci alone are easily changeable and fail to evaluate the vulnerability of EIE species.

In all comparisons between EIE and NE species, the EIE species showed higher proportions of non-synonymous variation to the total SNVs in transcripts with heterozygous loci, suggesting a decreased efficacy of selection as a result of decreased *N*e. The proportions of non-synonymous SNVs with deleterious impacts on protein function or loss of function were always higher in EIE species than in NE species. These results were unchanged when they were recalculated only for the non-duplicated transcripts. Although the significantly higher proportion of non-synonymous SNVs to the total SNVs and proportions of deleterious non-synonymous SNVs were not supported in endangered *A. boninsimae* than in non-endangered *A. pygmaea*, this result was likely owing to self-fertilization in both species. Furthermore, all EIE island species accumulated more deleterious variants at substitution sites than did NE species. Deleterious substitutions at homozygous sites are expected to reflect a long-term reduction in the efficacy of selection, and the results suggest that the EIE species have been under long-term weak purifying selection. The accumulation of deleterious variants seemed to be similar to that on heterozygous sites between *A. boninsimae* and *A. pygmaea*. However, the accumulations of deleterious mutations at the substitution sites suggested that the population of *A. boninsimae* has been maintained under weak purifying selection for a longer time than *A. pygmaea*. These results suggest that the colonization of the island or repeated self-fertilization in isolated populations of *A. boninsimae* have strongly affected the accumulation of deleterious mutations. Comparisons of the proportion of deleterious variations in both heterozygous and substitutional sites provide new insights into whether the threat of a limited gene flow occurred over an evolutionary or ecological time scale.

Although deleterious mutations at many loci will cause inbreeding depression and lower the fitness of species^7^, the predicted deleterious variants found in this study might partly include particular adaptive variations that were necessary to inhabit specific environments on these islands. That is, although some variants, which were predicted to be deleterious owing to the rarity of the variation in the existing protein database and expected large functional change in the protein, might increase the fitness of the plants in the local specific environment. However, variations resulting in premature stop codons can cause the loss of function in genes, and higher proportions of the nonsense mutations at non-synonymous sites suggest the decreased adaptability of EIE species. Thus, the present results strongly suggested that higher proportions of predicted deleterious mutations in EIE species were caused by weaker purifying selection, rather than natural selection increasing the frequency of favorable alleles.

*P*_D_ is known to have a positive correlation with the habitat variability the species experiences^33,34^. Although divergence time and phylogeny were also examined as to whether they could influence *P*_D_, no correlations were found^27^. A recent study showed that invasive species with high environmental adaptability had high *P*_D_^32^. In additionally, the loss of duplicated genes occurred more frequently in species with low *P*_D_^27^. Therefore, *P*_D_ is expected to have a relationship with species vulnerability, and should be suitable for the evaluation of low fitness in endangered species. EIE species generally demonstrated lower *P*_D_ than NE species. Although the lower *P*_D_ in EIE species was not significant in some species pairwise comparisons, the opposite trend was only found between *C. ameristophyllum* and *C. keiskeanum*; both species were only represented by one specimen. Comparisons based on additional sampling might support the lower *P*_D_ in endangered *C. ameristophyllum* than in non-endangered *C. keiskeanum*.

Unlike previous studies that identified a positive correlation between *P*_D_ and habitat variability of species based on whole-genome data, the estimated *P*_D_ values in this study were based on short-read *de novo* RNA-seq assembly. The values of some species showed large intraspecific variations (e.g., *C. grandicollum*), although the number of genes was not so variable within a species. Thus, some conditional differences in gene expression, analyzed tissues, or sequencing platforms may affect our estimates. Estimation of *P*_D_ must be examined using multiple specimens when it is conducted using RNA-seq. However, given that lower average *P*_D_ values were generally evident in the present EIE species compared to NE species, the genome of the range-restricted plant species may have evolved to have a lower *P*_D_, resulting in their potential vulnerability.

We compared the genome-wide genetic diversity, accumulation of deleterious variations, and *P*_D_ between EIE and the NE plant species of four genera based on exhaustive expressed gene transcripts. Most investigations met the theoretical expectations that EIE island species exhibited lower genetic diversity and *P*_D_ and accumulated deleterious variations in their functional loci. It is difficult to decipher if the present results can be attributed solely to the rarity of the species with regards to their small population size or also to various effects accompanied by their insularity. In this study, we have targeted the EIE species that must have regenerated with long-term restricted gene flow after a founder event. Thus, not all endangered species may experience the same genomic changes found with the present EIE species. However, these results suggested that population establishment and long-term isolation on islands is a critical factor to the loss of genetic diversity, and EIE species have thus been strongly affected by genetic drift and a reduction in the efficacy of selection due to severe population bottlenecks and limited gene flow in small populations. Thus, the newly investigated indices in our present study are valid for conservation of EIE species. Indeed, a large difference between the genomes of EIE and NE species would be evident due to the accumulation of deleterious variations in functional loci, especially deleterious variants on heterozygous loci that seem to reflect recent threats. These results not only provide new insights into how the genome changes in EIE species, but they also suggest that serious vulnerability of EIE species may be overlooked when assessing genetic diversity through neutral loci alone. Genetic diversity data are important for designing effective conservation and management programs, as genetic diversity is associated with the risk of extinction. Our results emphasize that the extinction risk of an EIE species cannot be evaluated by genetic diversity alone, even if the genome-wide genetic diversity is assessed.

Conservation and breeding projects in the Ogasawara Islands were promoted by the Ministry of the Environment, Japan in four of the seven presently EIE species (*A. boninsimae*, *C. grandicollum*, *C. hoshii*, and *M. tetramerum*). However, these projects have not yet achieved the successful propagation and conservation of these target species in the wild. Although the plants can be cultivated in botanical gardens, the seedlings are fragile in the wild; therefore, natural regeneration has not occurred in most cases^23^. For example, although the Koishikawa Botanical Garden, University of Tokyo began the ex situ conservation and vegetative propagation of *M. tetramerum* in 1983^45^, saplings replanted from the ex situ population cannot survive long-term in the wild, and the population continues to decline. These vulnerabilities may be attributed to the observed accumulation of deleterious mutations. Available resources and efforts toward biodiversity conservation are limited, and it is necessary to maximize the conservation efficiency through the effective allocation of resources. To achieve favorable results, genomic research could be useful for prioritizing species for conservation and selecting individuals or populations for replanting programs. In addition to practical projects, reviewing the conservation efficiency with results from genomic research is expected to improve conservation strategies.

## Methods

### RNA extraction, RNA-seq, and de novo assembly

Tissues (leaf, inflorescence, or bud) of all EIE species were sampled from plants cultivated from wild cuttings in Koishikawa Botanical Garden, University of Tokyo; the tissues of NE species were collected from the wild (Supplementary Data 3). Note that the EIE plants in the botanical garden have not experienced a change in generation since being collected. All tissue samples used in this study are preserved as voucher specimens at the Laboratory of Forest Biology, Graduate School of Agriculture, Kyoto University or the University of Tokyo (Supplementary Data 3). Total RNA was extracted from fresh or fixed tissues using RNAlater (Thermo Fisher Scientific, San Jose, CA, USA) with the help of an Agilent Plant RNA Isolation Mini Kit (Agilent Technologies, Santa Clara, CA, USA) according to the manufacturer’s instructions. The concentration of RNA was checked using a MultiNA microchip electrophoresis system (SHIMADZU, Kyoto, Japan). RNA samples derived from single individuals of a species were sequenced separately on Illumina HiSeq 1000, HiSeq 2500, HiSeq 4000, or HiSeq X sequencers at 90, 100, or 150 nucleotide paired-end (PE) reads (Supplementary Data 1). All paired comparisons were conducted between values derived from the same original read lengths, as the level of uniquely mapped reads can vary according to the read lengths^46^. Sequence processing and analysis were conducted on the supercomputer at the Research Organization of Information and Systems, National Institute of Genetics, Japan. In our pilot analysis, we found that the contamination of low-quality reads made it difficult to compare between the data obtained from different sequencing platforms, especially by HiSeq 1000. Therefore, we set a standard for low-quality reads, which occur when over 10% of the bases have a quality score < 30, were discarded by the FASTQ Quality Filter implemented in the FASTX-Toolkit (http://hannonlab.cshl.edu/fastx_toolkit/). Paired reads were only used in the reconstruction of transcript sequences. Two NE species of the genus *Ajuga* (*A. pygmaea*, and *A. shikotanensis*) used in this study were known to have polyploid genomes^47^. Because Trinity performed well when separating homoeologous copies originating from complex polyploid genomes^48^, Trinity ver. 2.2.0^49^ was used for de novo RNA-seq assembly.

### Homology search and duplicated gene identification

When splicing isoforms for transcripts were available, we used the longest transcript of the contigs that had the same accession with different clusters, genes, and isoforms in the output of Trinity assembly. We focused on the genes that might acquire some new function after duplication, rather than the simple copies of original genes. After identifying a reading frame for each longest contig, they were translated into amino-acid sequences. The sequences comprising less than 50 amino acids were excluded from further analysis. BLASTP searches were carried out through the NCBI-BLAST-2.4.0 + program using the amino acid sequences of the longest transcripts to identify duplicated genes in the plant materials. Transcripts sharing homology with other transcripts (*E* value < 10^–4^ and query coverage > 30%) were identified as candidate duplicated genes, and transcripts without any homology with other transcripts were identified as candidate singleton genes.

Protein sequences of all 39 angiosperm species from 10 orders registered in EnsemblPlants (release 30; http://plants.ensembl.org) were downloaded and used to create the plant protein database. Thirty-nine species were described in SI. Transcripts sharing homology with the above plant protein sequences were searched using BLASTP (*E* value < 10^–4^) through the NCBI-BLAST-2.4.0 + program. The transcripts that did not show homology with plant protein sequences were filtered out. Candidate duplicated and singleton genes that shared homology with other plant proteins were defined as duplicated genes and singleton genes, respectively. *P*_D_ was defined as the proportion of the number of duplicated genes relative to the total number of genes.

### Identification of heterozygous SNVs

To identify heterozygous SNVs within an individual, all reads were mapped by BWA version 0.7.13 (ref. ^50^) to the reference of all the transcripts reconstructed by Trinity. In the process of mapping by BWA, the options for mismatch penalty (-B) and minimum seed length (-k) were set 13 and 50, respectively, to avoid inaccurate SNVs. SNVs were identified by SAMtools version 1.4.1 (ref. ^51^). Only SNVs with minimum Root Mean Square mapping quality > 30 were output. After sorting heterozygous SNVs into synonymous or non-synonymous, the number of heterozygous synonymous SNVs, which were putative neutral variations, were calculated per kb of each longest transcript and representative of genetic diversity.

### Estimation of deleterious amino-acid variation

For the transcripts with homology with protein sequences of 39 plants in EnsemblPlants, we determine whether each amino acid variation on non-synonymous SNVs might affect protein function by PROVEAN^36^ was used, with the default threshold of a PROVEAN score prediction of deleterious variation equal to or below − 2.5, and the variant whose score is above − 2.5 is predicted to have a “neutral” effect. Although the original amino-acid variant of two heterozygous variants cannot be distinguished without phylogenetic analysis, it has been reported that most amino-acid changes are deleterious or neutral^31^. Thus, the non-synonymous variations whose PROVEAN scores were above |2.5| can be regarded as deleterious without distinguishing variants from references. In estimation using SIFT^37^, amino-acid variations whose SIFT prediction scores were below 0.05 were regarded as deleterious, and the proportion of deleterious amino acid variations of total non-synonymous SNVs were calculated in each estimation. The SNVs that could not be evaluated in SIFT were excluded from the calculation. We calculated the proportion of deleterious non-synonymous variants for each gene. In addition, we identified SNVs that varied by their nonsense SNVs (loss-of-function SNVs), and calculated the proportion of loss-of-function SNVs in the total non-synonymous SNVs for each species.

### Examination of indices by excluding candidate paralogs

Although options or quality thresholds were employed in read mapping and calling processes to avoid inaccurate SNVs, there remains the possibility that the number of heterozygous SNVs may be artifactually inflated on paralogous transcripts due to the difficulty in mapping. Thus, we also calculated the number of heterozygous synonymous SNVs, the proportion of non-synonymous SNVs to the total synonymous SNVs, and the proportion of deleterious non-synonymous variants for a set of longest transcript or genes that were excluding all candidate paralogs.

### Proportion of deleterious amino-acid substitutions during evolution

To identify the substitution sites between EIE and NE species and estimate the proportion of deleterious substitutions of those sites, we constructed a fresh set of integrated reference transcripts for each species by de novo assembly with all reads from multiple specimens. Then, orthologous gene transcripts between two species were identified by a BLASTP search with > 80% sequence identity. For this BLASTP search, the longest coding sequences were used when multiple isoforms with the same gene ID were reconstructed. Nucleotide substitutions in orthologous transcripts of EIE species and NE species were reciprocally identified. Heterozygous loci for transcripts in each species were excluded from this analysis, after which, we identified all intraspecies heterozygous variants by mapping all reads from multiple specimens to the integrated reference transcripts using BWA and SAMtools. The transcripts that did not show homology with plant protein sequences were also filtered out. The substitutions were categorized as synonymous or non-synonymous. Non-synonymous substitutions were additionally determined to be deleterious using PROVEAN and SIFT. In the results by PROVEAN, the positive scores include the impact of substitutions in species used as a database for the alignment of orthologous genes. We defined the variants whose PROVEAN score was below − 2.5 as deleterious in the species used as the query. The proportions of deleterious substitutions were calculated by dividing the number of non-synonymous substitutions with PROVEAN scores less than zero. Estimations of deleterious substitutions by PROVEAN and SIFT were all carried out with both EIE and NE species reciprocally used as queries. Proportions of deleterious substitutions were calculated for each gene.

### Statistics and reproducibility

In all estimations, when multiple specimens could be analyzed for one species, the mean value was used as the species value for the statistical comparisons. When only one specimen was available (i.e., *C. ameristophyllum* and *C. keiskeanum*), the value estimated for that specimen was treated as the species value. The following six indices were compared between the congenic EIE and NE species using *t* tests: *P*_D_, the number of heterozygous synonymous SNVs calculated per kb for the longest transcript, proportions of non-synonymous SNVs to the total number of SNVs calculated for each, the proportion of nonsense SNVs to total non-synonymous SNVs on transcripts with heterozygous loci, and the proportion of deleterious non-synonymous variants calculated for each gene by PROVEAN and SIFT. In addition, the mean values for the proportion of deleterious substitutions calculated for each gene by PROVEAN and SIFT were compared between the congenic EIE and NE species using *t* tests. All indices were recalculated excluding all candidate paralogs and were compared with the same statistical methods. Further, the overall trends of the above values between the EIE and NE species across the four genera were examined using exact binomial tests.

### Reporting summary

Further information on research design is available in the Nature Research Reporting Summary linked to this article.

## Supplementary information

Supplementary Information

Description of additional supplementary items

Reporting Summary

Supplementary Data 1

Supplementary Data 2

Supplementary Data 3

## Data Availability

All non-sequence data analyzed during this study are included in this manuscript (and its supplementary information files). Paired-end sequencing data obtained in this study have been submitted to the DDBJ Sequence Read Archive (DRA) (http://trace.ddbj.nig.ac.jp/dra/index_e.html) under accession numbers PSUB008171, PSUB008172, PSUB008173, PSUB008174, and PSUB008177.
