# Supplementary material for: Endangered island endemic plants have vulnerable genomes
Source: Commun Biol. 2019 Jun 27;2:244. doi: 10.1038/s42003-019-0490-7 (PMC6597543; doi:10.1038/s42003-019-0490-7)
Supplement: Supplementary file 1 — Supplementary Information [file 42003_2019_490_MOESM1_ESM.pdf]

Supplementary Table 2. *P*-values of t-test compared the mean values of the number of synonymous SNVs at non-duplicated transcripts with heterozygous loci (the counts per kb), the proportion of non-synonymous SNVs to total SNVs on non-duplicated transcripts with heterozygous loci, the proportion of nonsense SNVs to total non-synonymous SNVs on non-duplicated transcripts with heterozygous loci, and the proportion of deleterious variations in non-synonymous SNVs on non-duplicated transcripts with heterozygous loci estimated by PROVEAN and SIFT between endangered island endemic [EIE] and non-endangered [NE] species.

	A. boninsimae **[EIE]**
A. pygmaea **[NE]**	
Synonymous SNV	0.511
Nonsyn/Syn SNV	0.396
Loss of function	0.315
PROVEAN	**0.018**
SIFT	0.447
A. shikotanensis **[NE]**	
Synonymous SNV	**< 0.001**
Nonsyn/Syn SNV	**< 0.001**
Loss of function	**0.002**
PROVEAN	**< 0.001**
SIFT	**0.005**

	C. grandicollum **[EIE]**	*C. linguifolium* **[EIE]**
C. lanceolatum **[NE]**		
Synonymous SNV	0.580	0.261
Nonsyn/Syn SNV	**0.003**	0.054
Loss of function	0.064	0.092
PROVEAN	**0.003**	**0.008**
SIFT	0.065	0.051

	C. hoshii **[EIE]**
C. triplicata **[NE]**	
Synonymous SNV	**0.010**
Nonsyn/Syn SNV	**0.006**
Loss of function	0.431
PROVEAN	**< 0.001**
SIFT	**0.010**

	M. tetramerum **[EIE]**
M. candidum **[NE]**	
Synonymous SNV	**0.005**
Nonsyn/Syn SNV	**0.015**
Loss of function	**0.030**
PROVEAN	**< 0.001**
SIFT	**0.003**
