# Supplementary material for: Endangered island endemic plants have vulnerable genomes
Source: Commun Biol. 2019 Jun 27;2:244. doi: 10.1038/s42003-019-0490-7 (PMC6597543; doi:10.1038/s42003-019-0490-7)
Supplement: Supplementary file 2 — Description of additional supplementary items [file 42003_2019_490_MOESM2_ESM.docx]

**Description of additional supplementary items**

**Supplementary Data 1**

Transcriptomic datasets.

**Supplementary Data 2**

Mean values of the number of synonymous SNVs at non-duplicated transcripts with heterozygous loci (the counts per kb), the proportion of non-synonymous SNVs to total SNVs on non-duplicated transcripts with heterozygous loci, the proportion of ~~stop-codons~~ nonsense SNVs to total non-synonymous SNVs on non-duplicated transcripts with heterozygous loci, and the proportion of deleterious variations in non-synonymous SNVs on non-duplicated transcripts with heterozygous loci estimated by PROVEAN and by SIFT.

**Supplementary Data 3**

Voucher information about the samples of present study.
