# Supplementary material for: Endangered island endemic plants have vulnerable genomes
Source: Commun Biol. 2019 Jun 27;2:244. doi: 10.1038/s42003-019-0490-7 (PMC6597543; doi:10.1038/s42003-019-0490-7)
Supplement: Supplementary file 3 — Reporting Summary [file 42003_2019_490_MOESM3_ESM.pdf]

nature research

Corresponding author(s): Takashi Makino

Reporting Summary

Nature Research wishes to improve the reproducibility of the work that we publish. This form provides structure for consistency and transparency in reporting. For further information on Nature Research policies, see Authors & Referees and the Editorial Policy Checklist.

Statistical parameters

When statistical analyses are reported, confirm that the following items are present in the relevant location (e.g. figure legend, table legend, main text, or Methods section).

n/a	Confirmed	
☐	☒	The exact sample size (*n*) for each experimental group/condition, given as a discrete number and unit of measurement
☒	☐	An indication of whether measurements were taken from distinct samples or whether the same sample was measured repeatedly
☒	☐	The statistical test(s) used AND whether they are one- or two-sided<br>*Only common tests should be described solely by name; describe more complex techniques in the Methods section.*
☒	☐	A description of all covariates tested
☐	☒	A description of any assumptions or corrections, such as tests of normality and adjustment for multiple comparisons
☐	☒	A full description of the statistics including central tendency (e.g. means) or other basic estimates (e.g. regression coefficient) AND variation (e.g. standard deviation) or associated estimates of uncertainty (e.g. confidence intervals)
☐	☒	For null hypothesis testing, the test statistic (e.g. *F*, *t*, *r*) with confidence intervals, effect sizes, degrees of freedom and *P* value noted<br>*Give P values as exact values whenever suitable.*
☒	☐	For Bayesian analysis, information on the choice of priors and Markov chain Monte Carlo settings
☒	☐	For hierarchical and complex designs, identification of the appropriate level for tests and full reporting of outcomes
☒	☐	Estimates of effect sizes (e.g. Cohen's *d*, Pearson's *r*), indicating how they were calculated
☒	☐	Clearly defined error bars<br>*State explicitly what error bars represent (e.g. SD, SE, CI)*

Our web collection on statistics for biologists may be useful.

Software and code

Policy information about availability of computer code

Data collection	HiSeq 1000<br>HiSeq 2500<br>HiSeq 4000<br>HiSeq X
Data analysis	FASTX-Toolkit 0.0.13<br>Trinity ver. 2.2.0<br>NCBI-BLAST-2.4.0+<br>BWA version 0.7.13<br>PROVEAN 1.1.5<br>SIFT 6.0.1

For manuscripts utilizing custom algorithms or software that are central to the research but not yet described in published literature, software must be made available to editors/reviewers upon request. We strongly encourage code deposition in a community repository (e.g. GitHub). See the Nature Research guidelines for submitting code & software for further information.

Data

Policy information about availability of data

All manuscripts must include a data availability statement. This statement should provide the following information, where applicable:

- Accession codes, unique identifiers, or web links for publicly available datasets
- A list of figures that have associated raw data
- A description of any restrictions on data availability

Accession codes, unique identifiers, or web links for publicly available datasets

Field-specific reporting

Please select the best fit for your research. If you are not sure, read the appropriate sections before making your selection.

☐ Life sciences ☐ Behavioural & social sciences ☒ Ecological, evolutionary & environmental sciences

For a reference copy of the document with all sections, see nature.com/authors/policies/ReportingSummary-flat.pdf

Ecological, evolutionary & environmental sciences study design

All studies must disclose on these points even when the disclosure is negative.

Study description	We compared the genome-wide genetic diversity, proportion of duplicated genes, and accumulation of deleterious variations of six endangered island plants from four genera with those of their non-endangered widespread congeners. We focused on exhaustive sequences of expressed genes obtained by RNA-sequencing.
Research sample	For RNA-sequencing, we used samples in the Ogasawara Islands which are classified as endangered by the List of Threatened and Endangered Wildlife Species in Japan (Ajuga boninsimae, Crepidiastrum grandicollum, Crepidiastrum ameristophyllum, Crepidiastrum linguifolium, Melastoma tetramerum, and Calanthe hoshii). We chose their closely related non-endangered plants with wider habitat ranges (Ajuga pygmaea, Ajuga shikotanensis, Crepidiastrum lanceolatum, Crepidiastrum keiskeanum, Melastoma candidum, and Calanthe triplicata).
Sampling strategy	No sample-size calculation was performed.
Data collection	RNA samples derived from single individuals of a species were sequenced separately on Illumina Hiseq (NOVOGENE Co.,Ltd, BGI, and Macrogen).
Timing and spatial scale	RNA were extracted from leaf, inflorescence, or bud.
Data exclusions	Low-quality reads, which occur when over 10% of the bases have a quality score < 30, were discarded by the FASTQ Quality Filter implemented in the FASTX-Toolkit.
Reproducibility	We provide RNA-seq datasets in this study as publicly available ones in DRA (PSUB008171, PSUB008172, PSUB008173, PSUB008174, and PSUB008177), and thus all results are reproducible.
Randomization	Our datasets did not require the use of randomization.
Blinding	Our datasets did not require the use of blinding.

Did the study involve field work? ☒ Yes ☐ No

Field work, collection and transport

Field conditions	Temperate zone in Japan for sampling non-endangered plants
Location	Ogasawara islands
Access and import/export	We accessed the habitats by domestic public transport.
Disturbance	Only tissues of non-endangered plants were collected from fields. Tissues of all endangered species were sampled from the plants cultivated in Koishikawa Botanical Garden, and thus the field in the Ogasawara Islands was not disturbed.

Reporting for specific materials, systems and methods

Materials & experimental systems

n/a	Involved in the study	
☒	☐	Unique biological materials
☒	☐	Antibodies
☒	☐	Eukaryotic cell lines
☒	☐	Palaeontology
☒	☐	Animals and other organisms
☒	☐	Human research participants

Methods

n/a	Involved in the study	
☒	☐	ChIP-seq
☒	☐	Flow cytometry
☒	☐	MRI-based neuroimaging
